# Energy efficiency and coding of neural network

**DOI:** 10.3389/fnins.2022.1089373

**Published:** 2023-01-11

**Authors:** Shengnan Li, Chuankui Yan, Ying Liu

**Affiliations:** College of Mathematics and Physics, Wenzhou University, Wenzhou, China

**Keywords:** Hodgkin-Huxley neuronal model, neural network, energy efficiency, energy coding, information entropy

## Abstract

Based on the Hodgkin-Huxley model, this study explored the energy efficiency of BA network, ER network, WS network, and *Caenorhabditis elegans* neural network, and explained the development of neural network structure in the brain from the perspective of energy efficiency using energy coding theory. The numerical simulation results showed that the BA network had higher energy efficiency, which was closer to that of the *C. elegans* neural network, indicating that the neural network in the brain had scale-free property because of satisfying high energy efficiency. In addition, the relationship between the energy consumption of neural networks and synchronization was established by applying energy coding. The stronger the neural network synchronization was, the less energy the network consumed.

## 1. Introduction

Neural networks are widely studied. In the human brain, neurons are connected by synapses to form structurally complex and computationally efficient neural networks. In processing various sensory information, the brain consumes a large amount of metabolic energy (Kety, 1957). Data show that the weight of the mammalian brain accounts for only 2% of the total body weight, but consumes about 20% of the total metabolic energy (Rolfe and Brown, 1997). Moreover, more than 70% of the energy consumed in the cortex is used directly for neural signal processing within subcellular cortical circuits (Howarth et al., 2012). For example, the opening or closing of ion channels in APs and the release of neurotransmitters in synaptic transmission (Laughlin et al., 1998; Valente et al., 2016). This metabolic energy expenditure may be large enough to affect the design, function and evolution of the brain, suggesting that the brain has to operate with extremely high energy efficiency (Aiello and Wheeler, 1995; Niven and Laughlin, 2008). Therefore, it is important to study the energy efficiency of neural networks. In the past decades, many studies have revealed strategies used by the nervous system to improve energy efficiency, including optimizing ion channel dynamics (Alle et al., 2009; Schmidt-Hieber and Bischofberger, 2010), optimizing the number of channels on individual neurons and the number of neurons in neuronal networks (Schreiber et al., 2002; Yu and Liu, 2014), maintaining a stable somatic temperature to minimize the energy consumed by individual action potentials (Yu et al., 2012; Wang et al., 2015), sparse coding (Olshausen and Field, 2004; Lörincz et al., 2012; Yu et al., 2014), neurotransmitter release at synapses with low probability (Levy and Baxter, 2002; Harris et al., 2012), etc. In addition, the structure of neural networks must also evolve to meet high energy efficiency due to the limited total metabolic energy in the brain (Corty and Freeman, 2013). However, due to the sheer number of neurons and synapses and the complex structure of connections, the calculation of the energy efficiency of neural networks remains one of the long-term challenges of modern neuroscience.

In recent years, the ratio of information rate to energy consumption rate has been mostly used to evaluate energy efficiency. It describes how much effective information is delivered by the network for each unit of energy consumed. The method needs to calculate the amount of energy consumed and the amount of information transmitted by the neural network. Methods for calculating the energy consumption of neurons are constantly being developed. Currently, there are sodium ion quantity estimation method (Stein, 2002; Crotty et al., 2006), cable energy equation estimation method (Carter and Bean, 2009), equivalent circuit method (Moujahid et al., 2011), and energy function method (Wang et al., 2008; Torrealdea et al., 2009). For the calculation of information, Shannon’s information theory is mainly applied. The entropy was introduced to quantify the amount of information transmitted by the system (Aczél and Daróczy, 1975). In addition to the method of evaluating network efficiency by the ratio of information rate and energy consumption rate, the study by Yuan et al. (2019) also explored the relationship between network efficiency and synaptic density. It was shown that the network efficiency could be evaluated by the inverse of the product of the average shortest path length of the neural network and the synaptic density. The larger the reciprocal, the more efficient the network is.

Some studies have shown that neural networks follow simple design rules similar to those of other networks (Laughlin and Sejnowski, 2003). In the field of complex network research, Erdös and Rényi (1959) first proposed the ER network model, which was considered for a long time as the most appropriate network to describe real systems. With the development of information technology, Watts and Strogatz (1998) constructed an intermediate network between a regular network and ER network, namely WS network. Then Barabási and Albert (1999) proposed a BA network based on the form of degree distribution having power-law functions (Barabási et al., 1999). And some studies found that neural networks have small-world property (Bullmore and Sporns, 2009; Park and Friston, 2013). In addition, neural networks in certain regions of the brain have scale-free property (Eguíluz et al., 2005). This indicates that neural networks are also in line with the direction of complex network development when choosing the network structure. A neural system can be viewed as a network formed by a large number of neurons interconnected by nerve fibers. Understanding how neural networks are organized and evolve can be combined with knowledge from the field of complex networks.

Researchers have been keenly exploring the mysteries of the human brain, and neural networks have been a hot topic of research. The generation, encoding and transmission of neural network information are extremely energy-consuming at the physiological-metabolic level. Energy efficient coding is an important problem facing neural networks. The evolution of neural network structure can be seen as an energy efficient way of coding. In order to cope with the complex system environment, the effective information capacity of the network transmission should be maximized, while the energy consumed by the network during the transmission should be minimized. This is the key principle to be followed by neural networks in the evolution process, and also the basic principle to be observed by the brain in the cognitive process. Therefore, energy coding theory can be used to study the evolution of neural networks. The energy coding theory proposed by Wang et al. (2018) shows that the membrane potential of a neuron corresponds to the neural energy it consumes. Currently, in the field of neuroscience, several traditional coding theories are followed, such as the group coding theory studied by Amari and Nakahara (2005), the neural group coding theory studied by Purushothaman and Bradley (2005) and the coding theory that can represent dynamic information in neural systems studied by Natarajan et al. (2008). Energy coding theory is superior to these traditional coding theories. It can study the global neural coding of brain function from the energy characteristics of neuronal activity. In addition, energy coding has superposition, which brings great simplicity to computation and analysis (Wang et al., 2018). In this paper, we calculate the energy efficiency of the neural network of *Caenorhabditis elegans*, ER network, WS network and BA network, and use energy coding theory to explain the reason why the neural network has the current development trend. It is also shown that energy efficiency can be used to evaluate a network, which is of great significance for studying the development and evolution of neural networks.

The paper is organized as follows: Section “2 Models and methods” describes the Hodgkin-Huxley neuronal model, and introduces the calculation methods of energy consumption rate, information rate and energy efficiency of neural networks. Section “3 Simulation results and analysis” shows the results of the simulations. We calculate the energy consumption rate, information rate and energy efficiency of the BA network, ER network, WS network, and *C. elegans* neural network under different electric synaptic coupling strengths. In addition, the effects of time box length on the information rate of the networks and the effects of noise on the energy efficiency of the networks are discussed. Conclusions are made in Section “4 Conclusion”.

## 2. Models and methods

### 2.1. Neuron electrical model

The study is based on the classical HH model (Moujahid et al., 2011), as shown in the following differential equation:


(1) $$\begin{array}{l} C\frac{dV}{dt}=-g_{Na}m^{3}h(V-E_{Na})\\ \;\;\;\;-g_{K}n^{4}(V-E_{K})-g_{l}(V-E_{l})+I, \end{array}$$

(2) $$\frac{d\,m}{d\,t}=\alpha_{m}\,\left(V\right)\,\left(1-m\right)-\beta_{m}\,\left(V\right)\,m,$$

(3) $$\frac{d\,n}{d\,t}=\alpha_{n}\,\left(V\right)\,\left(1-n\right)-\beta_{n}\,\left(V\right)\,n,$$

(4) $$\frac{d\,h}{d\,t}=\alpha_{h}\,\left(V\right)\,\left(1-h\right)-\beta_{h}\,\left(V\right)\,h.$$

where *V* is the membrane potential of the neuron. *C* is the membrane capacitance. *I* is the external stimulation current.*g_Na_*, *g_K_*, and *g_l_* are the maximum conductance of each ion channel, respectively. *m*, *n*, and *h* are dimensionless variables indicating gating variables for potassium and sodium channels.*E_Na_*, *E_K_*, and *E_l_* are the reversal potentials of each ion channel of the neuron in the resting state, also known as the Nernst potential. Equations 5–10 are the rate functions of ion channel opening and closing, and these functions can describe the change in the proportion of open channels over time.


(5) $$\alpha_{m}\,\left(V\right)=\frac{0.1\,\left(V+40\right)}{1-e^{-\left(V+40\right)/10}},$$

(6) $$\beta_{m}\,\left(V\right)=4\,e^{-\left(V+65\right)/18},$$

(7) $$\alpha_{n}\,\left(V\right)=\frac{0.01\,\left(V+55\right)}{1-e^{-\left(V+55\right)/10}},$$

(8) $$\beta_{n}\,\left(V\right)=0.125\,e^{-\left(V+65\right)/80},$$

(9) $$\alpha_{h}\,\left(V\right)=0.07\,e^{-\left(V+65\right)/20},$$

(10) $$\beta_{h}\,\left(V\right)=\frac{1}{1+e^{-\left(V+35\right)/10}}.$$

The values of the relevant parameters in the HH model are shown in Table 1.

**TABLE 1:** Parameters in the HH model.

Parameter	Value	Parameter	Value	Parameter	Value	Parameter	Value
*C*	1μF/cm ^2^	*g*_*Na*_	120 mS/cm ^2^	*g*_*K*_	36 mS/cm ^2^	*g*_*l*_	0.3 mS/cm ^2^
*E*_*Na*_	50 mV	*E*_*K*_	−77 mV	*E*_*l*_	−54.5 mV	*V*_0_	−65 mV

### 2.2. The method of calculating neural network energy consumption rate and information rate

Based on the HH model, the paper (Moujahid et al., 2011) proposes a method to estimate the energy consumption of neurons from the perspective of neuronal equivalent circuits. The energy consumption is calculated as follows:


(11) $$E\,\left(t\right)=\frac{1}{2}\,C\,V^{2}+H_{N\,a}+H_{K}+H_{l}.$$

The left side of the equal sign of Eq. 11 indicates the total electrical energy accumulated in the neuronal circuit. The first term to the right of the equal sign is the energy stored by the capacitor in the circuit, and the last three terms indicate the energy of each ion power source. Since the electric power is equal to the product of the current and the electric potential, derivation of Eq. 11 with respect to time yields Eq. 12 as follows:


(12) $$\overset{\cdot }{E}\left(t\right)=C\,V\,\overset{\cdot }{V}+i_{N\,a}\,E_{N\,a}+i_{K}\,E_{K}+i_{l}\,E_{l}.$$

According to the first equation in the HH model


(13) $$C\,\overset{\cdot }{V}=-i_{N\,a}-i_{K}-i_{l}+I.$$

Where,


(14) $$i_{N\,a}=g_{N\,a}\,m^{3}\,h\,\left(V-E_{N\,a}\right),$$

(15) $$i_{K}=g_{K}\,n^{4}\,\left(V-E_{K}\right),$$

(16) $$i_{l}=g_{l}\,\left(V-E_{l}\right).$$

Substitute Eq. 13 into Eq. 12 to obtain Eq. 17,


(17) $$\overset{\cdot }{E}=V\,I-i_{N\,a}\,\left(V-E_{N\,a}\right)-i_{K}\,\left(V-E_{K}\right)-i_{l}\,\left(V-E_{l}\right).$$

Putting Eqs 14–16 into Eq. 17, we can get the energy consumption rate of change equation as,


(18) $$\begin{array}{l} \overset{\cdot }{E}=VI-g_{Na}m^{3}h{\left(V-E_{Na}\right)}^{2}\\ \;\;-g_{K}n^{4}{\left(V-E_{K}\right)}^{2}-g_{l}{\left(V-E_{l}\right)}^{2}. \end{array}$$

The first term on the right side of the equal sign in Eq. 18 represents the electrical power applied to the neuron by external stimulation, and the last three terms represent the electrical power of the ion channel, i.e., the energy consumed by the ion channel per second. Therefore, the energy consumed by the neuron can be calculated by Eq. 18.

**FIGURE 1:**
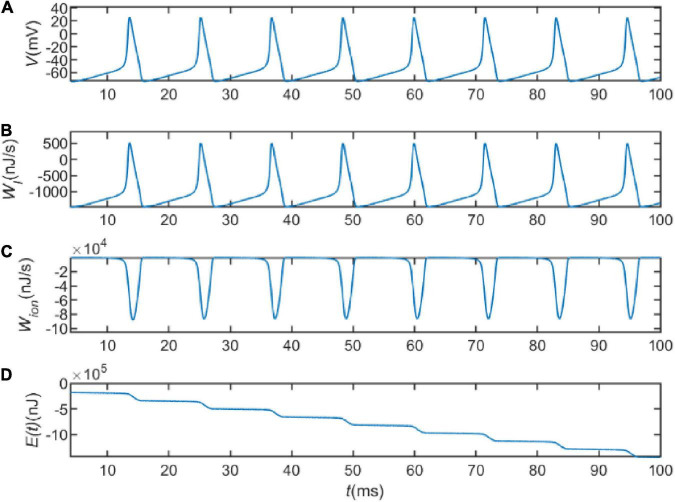
**(A)** The action potential delivered at *I* = 20 μA. **(B)** The electrical power provided by the external stimulus current. **(C)** The energy consumed per second by the ion channel. **(D)** The total energy consumed by the neuron.

Next, we explored the energy consumption of a neuron on action potentials. Figure 1A represents the sequence of action potentials generated by the neuron within 100ms in the presence of current *I* = 20 μA. The membrane potential changes from about −65to about 20 mV after depolarization. Figure 1B shows the variation of *W*_*I*_ = *VI*with time. It describes the energy provided or consumed per second by the external stimulation current, with a minimum value of about 1,400 *nJ*/s and a peak value of about 500 *nJ*/s. This suggests that the external stimulation current is sometimes energy-consuming and sometimes energy-providing. Figure 1C depicts the variation of the last three terms of Eq. 18 with time. Let


(19) $$\begin{array}{l} W_{ion}=-g_{Na}m^{3}h{\left(V-E_{Na}\right)}^{2}-g_{K}n^{4}{\left(V-E_{K}\right)}^{2}\\ \;\;\;\;\;-g_{l}{\left(V-E_{l}\right)}^{2}. \end{array}$$

which indicates the energy consumed per second by the ion channel. As can be seen in Figure 1C, the peak of the electrochemical energy consumption of the ion channel is nearly 90,000 nJ/s, which is much higher than the energy of *W*_*I*_. It indicates that the energy consumption of the whole circuit is mainly on the energy consumption of the ion channel. The consumed energy is provided by hydrolyzing ATP molecules to maintain neuronal activity. Figure 1D represents integration of Eq. 18 over the total time, depicting the variation of energy consumed by the neuron with time. By comparing the firing of neuronal action potentials, it can be found that the neuron consumes little energy during the resting state, but consumes a lot of energy when generating action potentials. This is due to the fact that the transmembrane movement of ions generates a large amount of metabolic energy consumption. Signals are propagated in neural networks with action potentials as carriers, indicating that the generation and transmission of information requires a large amount of metabolic energy. This also suggests that neural energy can encode neural signals.

Gap junctions are a form of direct intercellular communication between cells, and are special connectivity structures that exist between cells. Gap junctions are present in almost all tissues of the body, except for fully developed skeletal muscle and mobile cell types in adults. Gap junctions located in neurons are often referred to as electrical synapses (Moujahid et al., 2011). When two or more neurons are coupled together, electrical synapses are frequent and play an important role in the synchronization of cellular activity. In particular, they are very efficient in transferring and synchronizing the information of neural networks (García-Pérez et al., 2004).

The current at the gap junction of two neurons in a neural network can be expressed as *I*_*Junction*_ = *k*(*V*_*i*_−*V*_*j*_). Where the parameter *k* is the conductance at the connection or the coupling strength, in the unit of *mS*/*cm*^2^.*V_i_* and *V_j_* represent the membrane voltage of the *i_th_* neuron and the *j_th_* neuron in the neural network, respectively, in the unit of *mV*. The current at the gap junction is provided by a unity-gain amplifier, and the electrical power provided by the amplifier to the *j_th_* neuron in the neural network is $\sum_{i=1}^{n}C_{i\,j}\,V_{i}\,I_{J\,u\,n\,c\,t\,i\,o\,n}$. Therefore,


(20) $$\begin{array}{l} \overset{\cdot }{E_{j}}(t)=CV_{j}\overset{\cdot }{V_{j}}+i_{jNa}E_{Na}+i_{jK}E_{K}\\ \;\;\;\;\;+i_{jl}E_{l}+\sum_{i=1}^{n}C_{ij}V_{i}I_{Junction}. \end{array}$$

The equation for the membrane potential of the *j_th_* neuron in the neural network is


(21) C⁢Vj⋅j=Ij-ij⁢N⁢a-ij⁢K-ij⁢l+∑i=1nCi⁢j⁢IJ⁢u⁢n⁢c⁢t⁢i⁢o⁢n.

Substituting Eq. 21 into Eq. 20, the energy change rate formula of the *j_th_* neuron can be obtained as follows:


(22) $$\overset{\cdot }{\begin{array}{l} \overset{\cdot }{E_{j}}=V_{j}I_{j}-g_{Na}m_{j}^{3}h_{j}{\left(V_{j}-E_{Na}\right)}^{2}-g_{K}n_{j}^{4}{\left(V_{j}-E_{K}\right)}^{2}\\ \;\;\;-g_{l}{\left(V_{j}-E_{l}\right)}^{2}+\sum_{i=1}^{n}kC_{ij}V_{j}\left(V_{i}-V_{j}\right)\\ \;\;\;+\sum_{i=1}^{n}kC_{ij}V_{i}\left(V_{i}-V_{j}\right) \end{array}}$$

where *C* is the adjacency matrix of the neural network, and *n* is the number of neurons in the neural network. The first term on the right side of the equation represents the energy consumed by the external stimulation current of the *j_th_* neuron, and the last three terms represent the energy consumed by the ion channel per second. The last two terms correspond to the sum of the energy consumption at all connections with the *j_th_* neuron as the postsynaptic neuron, and they represent the energy consumed by the current at the gap junction and the energy provided by the amplifier, respectively. The connection is not necessarily consuming energy, it may also be supplying energy. The energy consumption rate of the whole neural network is *E_N_* = ⟨∑*_j_E_j_*⟩*_t_*. Where *E_j_* is the integral of the energy change rate over the total time, and ⟨⟩*_t_* represents the average over time *t*.

In order to quantify the amount of information transmitted in a neural network, the information entropy in Shannon’s information theory is used to estimate the information content of the neural network.

The specific calculation is performed using the method mentioned by Strong et al. (1996). Firstly, the relevant parameters of the neuron model are set, and the firing sequence of the neuron is obtained. The sequence is then placed into a time bin of length △*t*, and set to “1” if a release occurs in the box, and “0” if no release occurs. Then the sequence is transformed into a “word” of *l* characters with a sliding time window of size *T*, and the length of the “word” is$l=\frac{T}{△\,t}$. The non-overlapping time window *T* is slid over the entire firing sequence, resulting in a sequence of words represented by ω*_i_*. Finally, the probability of each word appearing in the sequence is counted, which is represented by *p*(ω_*i*_). Eq. 23 represents the information entropy rate.


(23) $$H^{T}=-\frac{1}{T}\,\sum_{i}p\,\left(\omega_{i}\right)\,\log_{2}p\,\left(\omega_{i}\right).$$

Calculate the information entropy rate of all neurons in the neural network using the above formula. The amount of information transmitted by the neural network per second is the sum of the information entropy rates of all neurons in the network, denoted by *I_N_*.

Finally, we define the energy efficiency of a neural network as the ratio of the information rate to the energy consumption rate, i.e., $\varepsilon_{N}=\frac{I_{N}}{E_{N}}$. It indicates how much effective information is delivered by the neural network for a unit of energy consumed.

## 3. Simulation results and analysis

This part is a simulation experiment. Three classical artificial networks, ER network, WS network, and BA network are selected for the experiment. The neural network of *C. elegans* is used as the reference network. Due to the large number of neurons in the organism and the complex structure of neural network connections, the research on general biological neural network is not thorough enough. In contrast, researchers have found that *C. elegans* possesses only about 300 neurons, and about 1,000 cells. And it also has a clearer structure of connections between neurons. Therefore, *C. elegans* is suitable for the study of biological neural network problems. The neural network structure of *C. elegans* has been largely explored, which is of great help for the study of neural network simulation and kinematic properties. In addition, it is capable of exhibiting learning, memory, exploration, and complex locomotion (Cho, 2011). The present artificial neural networks are still difficult to achieve these abilities. Therefore, the neural network of *C. elegans* is chosen as the reference network for the experiment.

There are 297 nodes in the experimentally selected neural network of *C. elegans*, and the network contains 2,345 connected edges. The adjacency matrices of the ER network, WS network, and BA network were obtained with reference to *C. elegans* neural network. The adjacency matrices of all networks are directed matrices with about 2,345 contiguous edges. In the following simulation experiments, we added the same external stimulus currents, all of which were uniform random numbers between 7 and 30μA.

### 3.1. Energy consumption rate

**FIGURE 2:**
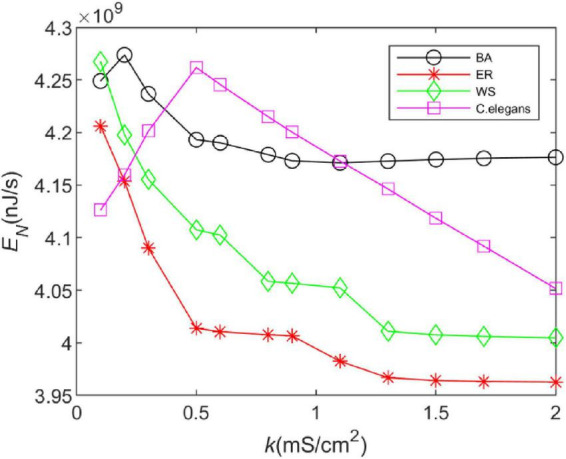
The energy consumption rate of the BA network, ER network, WS network, and *Caenorhabditis elegans* neural network at different coupling strengths. The external stimulus current is 7∼30μ*A*.

In this section, the energy consumption rates of the BA network, ER network, WS network, and neural network of *C. elegans* are calculated. We integrated Eq. 22 over the total time and took the opposite sign of the actual calculation to obtain the energy consumed by a single neuron. The energy consumed by the neural network is the sum of the energy consumed by 297 neurons, which is due to the linear superposition of the energy encoding. The total energy of the neural network after superposition can be used to reflect the synchronization state of the whole network. Therefore, the analysis of intricate perceptual cognition can be simplified from the perspective of neural energy (Wang et al., 2018). The energy consumption rate is the average of the total energy over the total time. The calculation time is 1,000 ms and the coupling strength is *k* ∈ [0.1,2]. The calculation results are shown in Figure 2.

From Figure 2, it can be found that the energy consumption of both the BA network and the *C. elegans* neural network gradually increases as the coupling strength increases, and decreases gradually after reaching a maximum value. However, with the increase of coupling strength, the energy consumption of the BA network tends to be stable, while that of the *C. elegans* neural network is still decreasing. The energy consumption rate of WS and ER network decreases with the increase of coupling strength, and the decreasing trend tends to be steady. And the ER network consumes less energy per unit of time. In contrast, it is found that the BA network and the neural network of *C. elegans* consume more energy. The high energy consumption represents that the network transmits more information, which can be reflected in the information rate in the next section.

**FIGURE 3:**
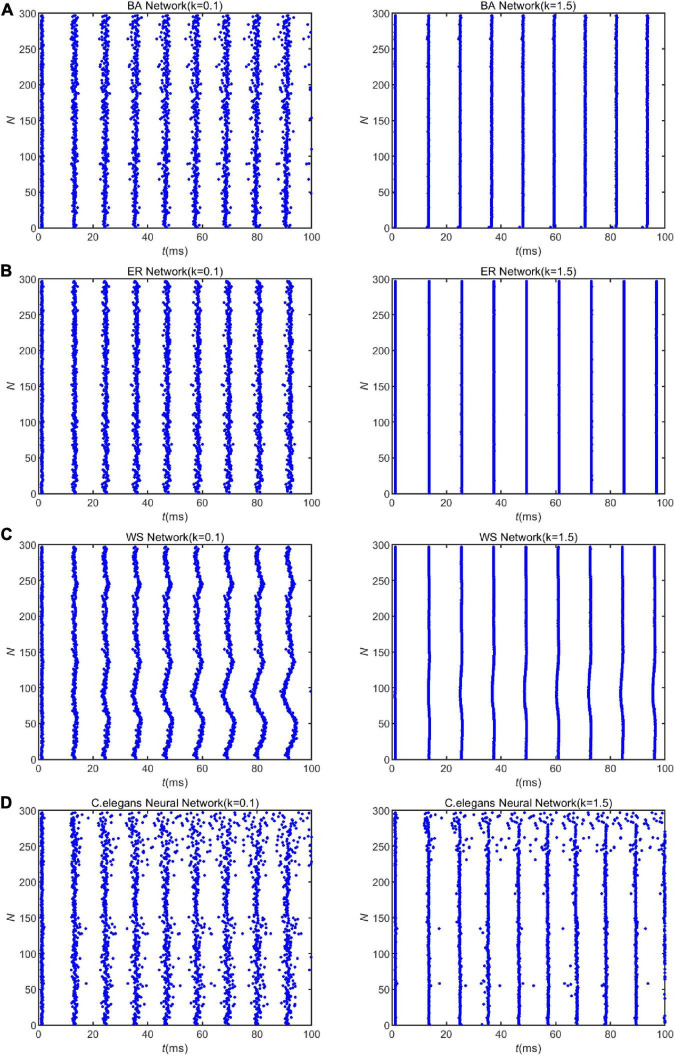
The spike record of the neural network with external stimulation current 7∼30μ*A*. Panels **(A–D)** show the spike record of the BA network, ER network, WS network, and *Caenorhabditis elegans* neural network within 100*ms*, respectively. The left side shows the spike record when the coupling strength is *k* = 0.1*mS*/*cm*^2^, and the right is *k* = 1.5*mScm*^2^.

Furthermore, Figure 2 shows that increasing the coupling strength appropriately can reduce the energy consumption of the neural network. This may be influenced by the synchronization of the network. Some experimental results have shown that thalamocortical neurons have high synaptic strength in early sleep and show well-synchronized activity, but in late sleep neurons show weak synchronization due to reduced synaptic strength (Esser et al., 2008). Since neuronal energy consumption can reflect the law of brain activity, the synchronization of neural networks is closely related to the energy consumption of the network. For this reason, we recorded the peak firing of the BA network, ER network, WS network, and neural network of *C. elegans* within 100 ms, as shown in Figure 3. The left side of Figure 3 indicates the peak firing of the four networks at coupling strength *k* = 0.1 *mS*/cm^2^ and the right side is *k* = 1.5 *mS*/cm^2^. The more stripy the record is, the more synchronized the network is. Therefore, it can be found from Figure 3 that the networks have stronger synchronization at higher coupling strengths. This is an intuitive prediction from the image, which cannot quantitatively estimate the network synchronization. Therefore, we chose the negative energy ratio, a synchronization index, to quantitatively analyze the synchronization of the network. The negative energy ratio can be used to describe the dynamic properties and energy encoding of the network, which helps to further explore the operation of the network (Zhu et al., 2018).

The negative energy ratio is defined as the ratio of the negative energy consumed by the network to the sum of the positive and energy consumed during the period from moment 0 to *t*, i.e.,


(24) $$\alpha \,\left(t\right)=\frac{E_{n\,e\,g\,a\,t\,i\,v\,e}}{E_{p\,o\,s\,i\,t\,i\,v\,e}+E_{n\,e\,g\,a\,t\,i\,v\,e}}\times 100\%,$$

(25) $$E_{n\,e\,g\,a\,t\,i\,v\,e}=\sum_{j=1}^{n}\int_{0}^{t}P_{j}\,\left(t\right)\cdot s\,g\,n\,\left(-P_{j}\,\left(t\right)\right)\,dt,$$

(26) $$E_{p\,o\,s\,i\,t\,i\,v\,e}=\sum_{j=1}^{n}\int_{0}^{t}P_{j}\,\left(t\right)\cdot s\,g\,n\,\left(P_{j}\,\left(t\right)\right)\,dt.$$

where *P_j_* (*t*)is the energy consumption power of the *j_th_* neuron at time *t*. The integral of *P_j_*(*t*) during [0,*t*]represents the energy consumed by the neuron. *sgn*(*x*) is the sign function defined as $sgn(x)=\{\begin{array}{c} 1,x>0\\ 0,x\leq 0 \end{array}.$ *E_negative_* and *E_positive_* represent the negative and positive energy consumed by the neural network in[0,*t*], respectively.

We re-expressed Eq. 22 as Eq. 27, as follows:


(27) $$\begin{array}{l} {\overset{\cdot }{\overset{\cdot }{E_{j}}=V_{j}I_{j}+P}}_{jNa}+P_{jK}+P_{jl}-V_{j}\left(i_{jNa}+i_{jK}+i_{jl}\right)\\ \;\;\;+\sum_{i=1}^{n}kC_{ij}V_{j}\left(V_{i}-V_{j}\right)+\sum_{i=1}^{n}kC_{ij}V_{i}\left(V_{i}-V_{j}\right). \end{array}$$

The electrical power consumed at the ion channel is divided into two parts, *P_jNa_* + *P_jK_* + *P_jl_* is the power of the voltage source represented by the Nernst potential (reversal potential). The other part *V_j_* (*i_jNa_* + *i_jK_* + *i_jl_*) represents the electrical power consumed driven by the membrane potential gradient (electric field force), which can be considered as the power of passive transport. The ion pump, during the operation, transports three sodium ions out of the cell and two potassium ions into the cell from the membrane (Crotty et al., 2006; Corty and Freeman, 2013). The ion pump constantly transports ions, which directly consume biological energy. Since the power represented by *P_jNa_* + *P_jK_* + *P_jl_* is approximately equal to the biological power of the ion pump, the power consumed by the ion pump can be calculated using the electrical power represented by the Nernst potential. It can be considered that the voltage source represented by the Nernst potential of the sodium ion is storing energy, while the reverse voltage source such as the potassium ion is consuming energy. We calculated the energy consumption by considering that this part is only consuming energy (Zhu et al., 2018), while in the calculation of the negative energy ratio *P_jNa_* + *P_jK_* + *P_jl_* is expressed as the following Eq. 28.


(28) $$P_{j}=P_{j\,N\,a}+P_{j\,K}+P_{j\,l}=\left|i_{j\,K}\,E_{K}\right|+\left|i_{j\,l}\,E_{l}\right|-\left|i_{j\,N\,a}\,E_{N\,a}\right|.$$

Applying Eq. 28 and the formula of negative energy ratio, we obtained the negative energy ratios of the BA network, ER network, WS network, and *C. elegans* neural network within 100 ms. The results are shown in Table 2. Combining with Figure 3, it can be found that when the coupling strength is larger, the negative energy ratio of the BA network, ER network, and WS network is smaller, while the negative energy ratio of the neural network of *C. elegans* is larger. It can be found that the negative energy ratios of the actual biological and artificial networks are differently influenced by the coupling strength. But all four networks show strong synchronization at higher coupling strengths. In summary, when the coupling strength is high, the synchronization of the network is strong, and the network consumes less energy. Therefore, the energy consumption of the network can be reduced by increasing the coupling strength appropriately.

**TABLE 2:** The corresponding negative energy ratio for Figure 3.

	k = 0.1 m*S*/cm^2^	k = 1.5 m*S*/cm^2^
BA	2.2839%	2.0860%
ER	2.2621%	2.1242%
WS	2.2581%	2.1032%
*Caenorhabditis elegans*	2.3329%	3.3974%

### 3.2. Information rate

**FIGURE 4:**
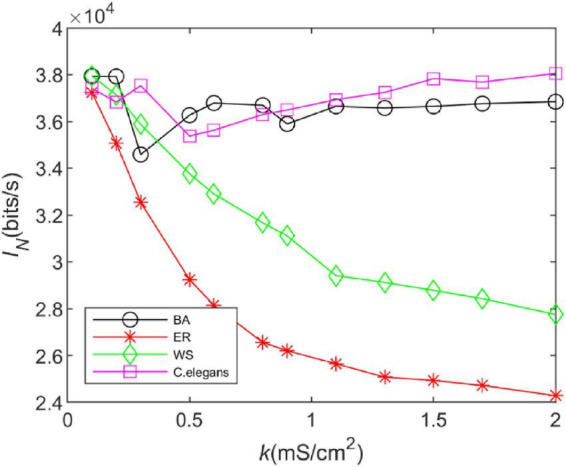
The information rate of the BA network, ER network, WS network, and *Caenorhabditis elegans* neural network at different coupling strengths. The external stimulus current added is 7∼30μ*A*.

According to Eq. 23, we analyzed the effect of coupling strength on the information rate of different neural networks. The total release duration is set to 9,000 *ms*, the time box length to △*t* = 3 *ms*, and the sliding time window length to *T* = 30 *ms*. The presence or absence of spikes in one of the boxes was coded as 1 or 0, respectively, so that each window represents a symbol in the binary alphabet. To estimate the amount of information transmitted by the network, 300 “words” were obtained experimentally. The calculation results are shown in Figure 4.

From Figure 4, it can be seen that the trend of the information rate of the BA network is basically the same as that of the *C. elegans* neural network. After a decreasing trend, the information rate increases steadily with the increase of the coupling strength. The trend of the information rate of the WS and ER networks with the coupling strength is the same. The higher the coupling strength is, the smaller the information rate is. In addition, the information rate of WS network is higher than that of ER network. BA network and *C. elegans* neural network transmit much more information per unit of time than WS and ER network. Since both the BA network and the *C. elegans* neural network have the scale-free property, but WS and ER network do not, it indicates that the connection structure of the network determines the information transfer function of the network. The network with scale-free property transmits more information per unit of time.

### 3.3. Energy efficiency

**FIGURE 5:**
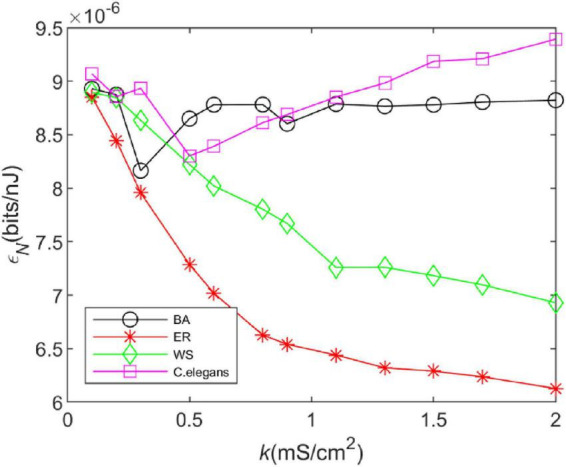
The energy efficiency of the BA network, ER network, WS network, and *Caenorhabditis elegans* neural network at different coupling strengths. Energy efficiency is defined as the ratio of information rate and energy consumption rate, i.e., $\varepsilon_{N}=\frac{I_{N}}{E_{N}}$.

Figure 5 depicts the effect of coupling strength on the energy efficiency of the neural network. The change in the energy efficiency of the BA network is more similar to that of the *C. elegans* neural network when the coupling strength *k*increases. The energy efficiency decreases gradually at first and increases gradually after producing a minimum value. The difference is that when the coupling strength is larger, the energy efficiency of the *C. elegans* neural network still shows an increasing trend, while the energy efficiency of BA network hardly changes anymore. WS network and ER network have lower energy efficiency, and the larger the coupling strength is, the lower the energy efficiency is. Compared to Figure 4, the energy efficiency of networks has roughly the same trend as the information rate, which indicates that the information rate is the main factor to determine their energy efficiency.

From the above results, it can be seen that the BA network transmits more information per unit of time and is more energy efficient compared to the WS and ER network. Therefore, in order to ensure the efficient operation of the brain, some neural networks in the brain will have scale-free properties. The theory of energy coding is used to link energy efficiency and network structure evolution, and the evolution direction of neural network structure is explained from the perspective of energy efficiency.

### 3.4. Parameter impact

#### 3.4.1. Time box length Δ*t*

The information entropy depends on the time box length △*t*. Therefore, we explored the effect of the time box length on the information rate of the neural network. The length of the “word” is set to 10, the length of the time box is △*t* ∈ [1:1:10], and the length of the sliding time window is *T* ∈ [10:10:100]. By adjusting the calculation time, 300 “words” were obtained for each calculation. The information rate of the *C. elegans* neural network, BA network, WS network, and ER network were calculated. The coupling strength at the neuron gap connection is set to *k* = 1.5 *mS*/cm^2^ at this time.

**FIGURE 6:**
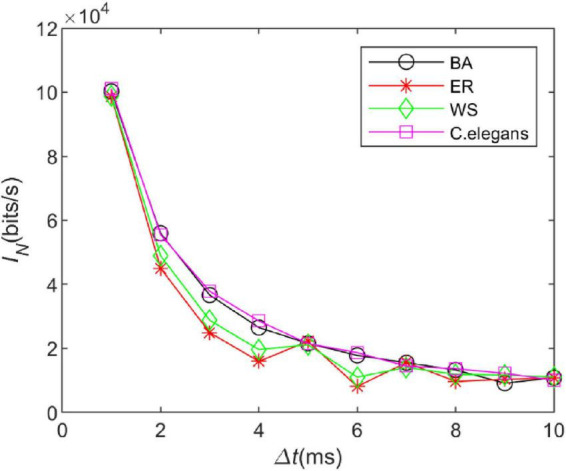
The information rate of the neural network at different time box lengths. The coupling strength at the gap junction is *k* = 1.5 *mS*/*cm*^2^. The external stimulus current added is 7∼30μ*A*.

In Figure 6, it is observed that as the length of the time box △*t*increases, the trend of the information rate of the four networks is basically similar and the information rate gradually decreases in all of them. The choice for △*t*in the calculation of the information rate of the issuance sequence is arbitrary. Since an accurate measurement of the issuance time can yield more information rate, for this reason, it is expected that a smaller time box length conveys a larger amount of information rate. The information rate does increase with decreasing △*t*as can be seen in Figure 6. We chose △*t*to be small enough so that the issued sequence can be converted into a binary sequence. It also has to be large enough to ensure that a good enough “word” sequence is obtained.

#### 3.4.2. Noise *I*_*noise*_

The actual network environment is affected by noise, and it is necessary to explore the effect of noise on the energy efficiency of the network. To better simulate the signal transmission environment of neurons in the network, we added Gaussian white noise with a mean of 0 and a variance of 1. The added noise is denoted by *I_noise_* in μ*A*. At this point, the first equation of the HH model becomes


(29) $$\begin{array}{l} C\frac{dV}{dt}=-g_{Na}m^{3}h\left(V-E_{Na}\right)-g_{K}n^{4}\left(V-E_{k}\right)\\ \;\;\;\;-g_{l}\left(V-E_{l}\right)+I+I_{noise}. \end{array}$$

**FIGURE 7:**
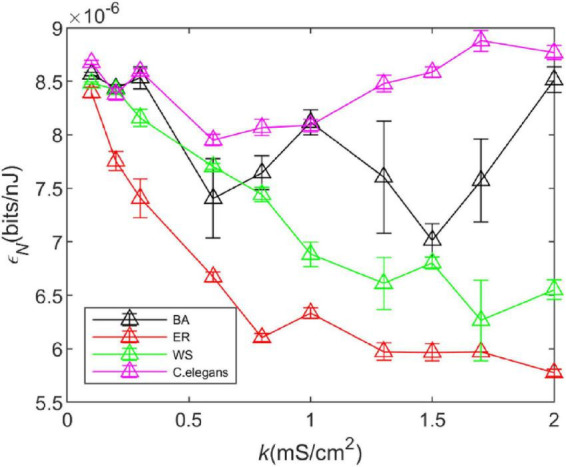
The energy efficiency of the BA network, ER network, WS network, and *Caenorhabditis elegans* neural network at different coupling strengths *k* when Gaussian white noise with mean 0 and variance 1 is added. Take the standard deviation of the results of five experiments. The calculation time of energy consumption rate is 1,000*ms*, and the calculation time of information rate is 1,500 *ms*. The external stimulus current is 7∼30μ*A*.

We took the standard deviation of the results of five experiments, as shown in Figure 7. It can be observed that the noise has little effect on the trend of energy consumption rate of the WS network, ER network, and *C. elegans* neural network with coupling strength, however, it has a great influence on that of BA network. It can be found that the energy efficiency of the BA network fluctuates greatly after adding noise. But the relationship between the energy efficiency of the BA network and *C. elegans* neural network and that of the WS and ER network has not changed.

**FIGURE 8:**
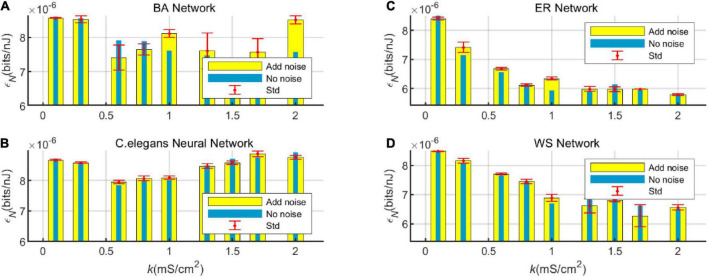
Bar graph of the energy efficiency of the BA network **(A)**, ER network **(C)**, WS network **(D)**, and *Caenorhabditis elegans* neural network **(B)** with and without white Gaussian noise.

In order to compare the change in energy efficiency of the network with and without noise more visually, the energy efficiency is shown in a bar graph, as shown in Figure 8.

Figure 8 visualizes the effect of noise on the energy efficiency of the network. From the graph, it can be found that at certain values of coupling strength, adding noise can improve the energy efficiency of the network, while sometimes noise can reduce the energy efficiency of the network. However, the noise has little effect on the overall trend of network energy efficiency with coupling strength. It can also be seen from the standard deviation that the fluctuation of the network energy efficiency by the noise is less. Therefore, the experimental model has good stability.

## 4. Conclusion

We investigated the energy efficiency of the BA network, ER network, WS network, and *C. elegans* neural network based on the HH neuron model. Energy efficiency is defined as the ratio of the information rate and energy consumption rate. Also, a method to calculate the energy consumption of the neural network is constructed. We found that the energy efficiency of the BA network was higher and closer to that of the neural network of *C. elegans*. This is consistent with the fact that neural networks in the brain have scale-free properties (Eguíluz et al., 2005), which can indicate that the evolutionary process of the brain satisfies high energy efficiency (Aiello and Wheeler, 1995; Niven and Laughlin, 2008).

We used energy coding and energy efficiency to further elucidate the effect of network structure on brain evolution. The relevant conclusions were also obtained in the study.

When exploring the energy consumption of neurons, more energy is consumed in generating action potential delivery and less energy is consumed in the resting state. Since information is transmitted in the neural network in terms of action potentials, this suggests that neurons consume a lot of energy when processing information. There is a correspondence between the energy consumption of the neural network and the synchronization of the network. The stronger the synchronization, the less energy the neural network consumes.

Comparing the trend of energy consumption rate, information rate and energy efficiency of the networks affected by the coupling strength, it can be found that the energy efficiency is mainly determined by the information rate. Since the BA network and the neural network of *C. elegans* have scale-free characteristics, their information rates are much higher than those of the WS and ER network. It indicates that the connection structure of the networks has a great influence on the information transfer function of the networks.

We also considered the effects of time box length and noise. It was found that the information rate of the neural network was higher when the time box length was shorter. In addition, the effect of Gaussian white noise on the energy efficiency of the neural network was explored. Gaussian white noise with a mean value of 0 and a variance of 1 was added, and the stability of the model was better at this noise intensity.

In summary, our study shows that the BA network has higher energy efficiency and is consistent with the requirement that the structural evolution of the neural networks in the brain should meet high energy efficiency. Our work explains why, in terms of energy efficiency, the evolutionary direction of neural networks in the brain selects for scale-free properties, and may contribute to further understanding of the structure and evolution of neural networks in the brain.

## Data availability statement

The original contributions presented in this study are included in the article/supplementary material, further inquiries can be directed to the corresponding author.

## Author contributions

SL: modeling and simulation. CY and SL: design and result analysis. SL and YL: writing and modification of the manuscript. All authors contributed to the article and approved the submitted version.
